# Coronary circulating mononuclear progenitor cells and soluble biomarkers in the cardiovascular prognosis after coronary angioplasty

**DOI:** 10.1111/jcmm.14336

**Published:** 2019-05-08

**Authors:** Juan Antonio Suárez‐Cuenca, Rogelio Robledo‐Nolasco, Marco Antonio Alcántara‐Meléndez, Luis Javier Díaz Hernández, Eduardo Vera‐Gómez, Alejandro Hernández‐Patricio, Karla Susana Sánchez‐Díaz, Juan Ariel Buendía‐Gutiérrez, Alejandra Contreras‐Ramos, Atzin Suá Ruíz‐Hernández, Rebeca Pérez‐Cabeza de Vaca, Paul Mondragón‐Terán

**Affiliations:** ^1^ Laboratory of Experimental Metabolism and Clinical Research, División de Investigación Centro Médico Nacional “20 de Noviembre” ISSSTE Mexico City Mexico; ^2^ Internal Medicine Department, HGZ 58 “Gral. Manuel Ávila Camacho” IMSS Mexico City Mexico; ^3^ Hemodynamics Unit, Cardiology Department Centro Médico Nacional “20 de Noviembre” ISSSTE Mexico City Mexico; ^4^ Laboratorio de Biología del Desarrollo y Teratogénesis Experimental Hospital Infantil de México Federico Gómez Mexico City Mexico

**Keywords:** EPCs, MACEs, Malondialdehyde, MMP‐9, sICAM‐1, SOD

## Abstract

Currently, there are no confident prognostic markers in patients with coronary artery disease (CAD) undergoing angioplasty. The present study aimed to explore whether basal coronary circulating Mononuclear Progenitor Cells (MPCs) and vascular injury biomarkers were related to development of major adverse cardiovascular events (MACEs) and may impact clinical prognosis.

**Methods:**

The number of MPCs and soluble mediators such as IL‐1β, sICAM‐1, MMP‐9, malondialdehyde, superoxide dismutase and nitric oxide were determined in coronary and peripheral circulation. Prognostic ability for MACEs occurring at 6 months follow up was assessed by time‐to‐event and event free survival estimations.

**Results:**

Lower coronary circulating MPCs subpopulations CD45^+^CD34^+^, CD45^+^CD34^+^CD133^+^CD184^+^, lower MMP‐9 and higher sICAM‐1 significantly associated with MACEs presentation and showed prognostic ability; while peripheral blood increase in malondialdehyde and decreased superoxide dismutase were observed in patients with MACEs.

**Conclusion:**

Coronary concentration of biomarkers related with vascular repair, such as MPCs subpopulations and adhesion molecules, may predict MACEs and impact prognosis in patients with CAD undergoing angioplasty; whereas peripheral pro‐oxidative condition may be also associated.

## 1 INTRODUCTION

Coronary ischemic injury induces early vascular repair mechanisms involving mobilization of cells with vascular repairing abilities like Mononuclear Progenitor Cells (MPCs) and production of soluble mediators of inflammation, remodelling and oxidative stress; with potential role as predictors of adverse cardiovascular events in patients with coronary artery disease (CAD) submitted to coronary angioplasty.^1^, ^2^ Intracoronary circulating biomarkers have been postulated as convenient, as they closer reflect vascular damage and repair.^3^, ^4^ However, characterization of coronary biomarkers in human studies has been scanty.^4^, ^5^ This study aimed to explore whether coronary MPCs and vascular injury biomarkers were related to the development of major adverse cardiovascular events (MACEs) and may have prognostic role in patients submitted to coronary angioplasty.

## 2 MATERIALS AND METHODS

### 2.1 Study design

Quasi‐experimental, correlational, prospective and observational study, that complied with the ethical guidelines of the 1975 Declaration of Helsinki and approved by the local Institutional Committees (096.2014), registered ClinicalTrials.gov NCT03583047. All participants provided written informed consent.

### 2.2 Study population and data collection

Fifty‐two consecutive candidates for coronary angiography, between March 2015 and February 2017, because of current CAD (chronic stable angina and acute coronary syndrome, including unstable angina, STEMI and NSTEMI). Due to methodological strategy to study variables interactions, and considering 95% CI alpha = 0.05, our sample size render a beta of 0.32; suggesting a careful interpretation of data”.

Clinical‐Demographic data were collected during study enrollment. High blood pressure, dyslipidemia and Diabetes Mellitus were defined according to JNC8, NCEP/ATPIII and ADA guidelines, respectively.^8^, ^9^

### 2.3 Coronary angiography, stenting and sample collection

Coronary angiography and stenting (everolimus or zotarolimus‐eluting stents, mean of 1 stent/vessel; 3 mm diameter, 20 mm length). Patients received standard dual antiplatelet therapy previous to Percutaneous Coronary Intervention (PCI) and antiplatelet therapy continued for at least 1 year. For blood sampling, 5 mL coronary blood was collected before angioplasty from the closest location to the atheroma plaque. Brachial peripheral blood sample was also collected.

### 2.4 Determination of circulating MPCs

Blood sample was submitted to density gradient to obtain lymphocytes, which were washed and fixed in 4% paraformaldehyde. Flow cytometry analysis (MACS Quant Analyser 10 ‐ Miltenyi Biotec) was performed within 12 hours in 1,000,000 events collected for each analysis. MPCs subpopulations were identified by their cell surface markers (CD45[lymphocytes], CD34[progenitor], KDR[VEGFR‐2,membrane marker of endothelial cells], CD133[early haematopoietic progenitor cells] and CD184[haematopoietic stem cells and endothelial cells]).

### 2.5 Measurement of plasma soluble biomarkers

sICAM‐1, superoxide dismutase (ELISA kits Human sICAM‐1 ThermoScientific and Human SOD‐1 Abcam, USA) and nitric oxide (Total Nitric Oxide and Nitrate/Nitrite Parameter Assay Kit, R&D Systems) were determined. IL‐1β was determined by immuno‐magnetic multiplexing assay, (MILLIPLEX MAP Human TH17 Magnetic Bead Panel) and Malondialdehyde (MDA) by a published method based on thiobarbituric acid.

### 2.6 Follow up and study endpoints

Follow‐up was conducted every month during 6 months, either via telephone interview or written reports from programmed medical evaluations. If telephone contact was not possible or physician visit were delayed >2 months, the endpoints were verified by an authorized person previously assigned. Primary endpoint consisted of MACEs, including: (a) cardiovascular death, (b) new myocardial infarction; (c) unstable angina, prompting to unscheduled visits to an emergency department within 24 hours, (d) episodes of decompensate heart failure requiring hospital attention. Secondary end points included prognostic markers, such as the time to present MACEs (dichotomized at 3 months cutoff) and the number of cases with MACEs‐free survival.

### 2.7 Statistical analysis

Data distributions were estimated using Kolmogorov–Smirnov test. Independent (1‐tailed) Mann‐Whitney or *t* test were applied for means comparison. Kaplan‐Meier curves and Log Rank analysis were performed for survival analysis. SPSS v.23 (SPSS Inc, IL,USA) was used for statistical analysis, where *P* ≤ 0.05 was considered significant.

## 3 RESULTS

The study population was constituted by 52 patients, mean aged 67.6 years old (31 males, 21 females) whose demographic and clinical characteristics are summarized in Table 1. (Table 1, upper panel). High blood pressure and dyslipidemia were significantly prevalent. Coronary angiography was indicated due to chronic stable angina in most of the patients. Lower coronary MMP‐9 was observed as compared with its peripheral concentration.

**Table 1:** Clinical‐demographic characteristics

	All (n = 52)	w/o MACEs (n = 32)	MACEs (n = 20)
Age (years old)	67.6 ± 8.59	66.8 ± 9.2	67.8 ± 9.1
Males	31 (59.6)	18 (56.2)	13 (65.0)
High blood pressure	37 (71.1)	25 (78.1)	12 (60.0)
Dyslipidemia	39 (75.0)	23 (71.8)	16 (80.0)
Diabetes mellitus	28 (53.8)	18 (56.2)	10 (50.0)
Active smoker	22 (42.3)	12 (37.5)	10 (50.0)
Weight (kg)	83.7 ± 14.7	85.5 ± 15.5	81.4 ± 13.7
Height (m)	1.70 ± 0.1	1.71 ± 0.1	1.68 ± 0.1
BMI (kg/m^2^)	28.6 ± 5.5	28.5 ± 4.2	29.0 ± 6.2
Total cholesterol (mg/dL)	246.6 ± 84.1	237.2 ± 87.9	260.8 ± 89.6
Triglycerides (mg/dL)	248.8 ± 82.3	251.8 ± 82.3	244.1 ± 85.2
Type of Angina			
*Chronic stable angina*	26 (50.0)	18 (56.3)	8 (40.0)
*Recent Myocardial Infarction*	14 (26.9)	8 (25.0)	6 (30.0)
*Unstable angina*	9 (17.3)	5 (15.6)	4 (20.0)
*Acute Myocardial Infarction*	3 (5.8)	1 (3.1)	2 (10.0)

MPCs populations (%)		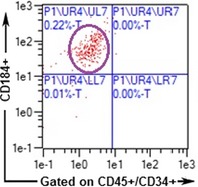	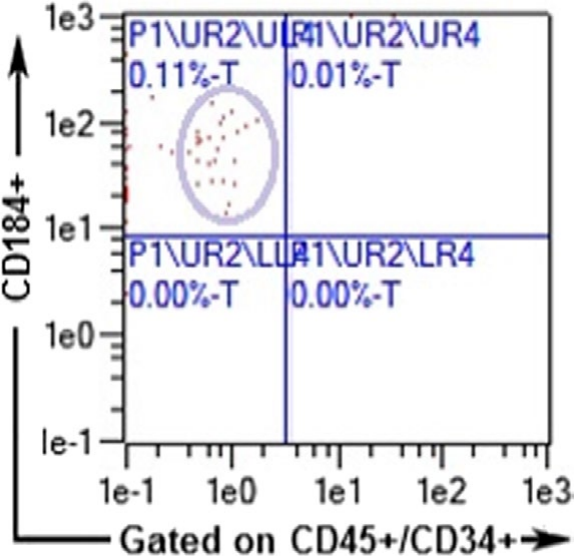
CD45^+^CD34^+^CD133^+^	0.06 ± 0.092	0.08 ± 0.03	0.03 ± 0.01^a^
CD45^+^CD34^+^CD184^+^	0.14 ± 0.025	0.16 ± 0.03	0.10 ± 0.04^a^
CD45^+^CD34^+^CD133^+^CD184^+^	0.007 ± 0.002	0.01 ± 0.003	0.001 ± 0.001^a^
CD45^+^CD34^+^KDR(VEGFR2) ^+^	0.012 ± 0.005	0.005 ± 0.002	0.02 ± 0.01
CD45^+^CD34^+^KDR(VEGFR2) ^+^CD184^+^	0.016 ± 0.007	0.020 ± 0.010	0.004 ± 0.003^a^
CD45^+^CD34^+^KDR(VEGFR2)^+^CD133^+^	0.023 ± 0.008	0.03 ± 0.01	0.02 ± 0.01

Vascular injury biomarkers	Coro	Periph	Coro	Periph	Coro	Periph
sICAM‐1 (pg/mL)	20.0 ± 6.3	19.8 ± 6.1	18.7 ± 4.9	17.9 ± 3.0	23.9 ± 8.6^b^	25.6 ± 9.7^b^
IL‐1β (ng/mL)	1.2 ± 0.87	1.4 ± 1.11	1.2 ± 0.75	1.3 ± 0.94	1.2 ± 1.15	1.9 ± 2.02
MMP‐9 (pg/mL)	91 ± 50	189 ± 149^a^	113 ± 59	208 ± 165	66 ± 18^a^, ^b^	132 ± 73
Malondialdehyde (ng/mL)	33.7 ± 8.4	36.2 ± 7.3	32.4 ± 8.1	32.3 ± 7.4	35.7 ± 9.5	43.3 ± 7.8^b^
Superoxide Dismutase (pg/mL)	3.1 ± 1.15	3.0 ± 1.59	3.4 ± 1.01	3.1 ± 1.59	2.8 ± 1.32	1.4 ± 1.10^b^
Nitric Oxide (µmol/L)	256 ± 103	245 ± 119	254 ± 114	259 ± 123	259 ± 93	206 ± 112

% MPCs are expressed as mean ± *SE* and the values of vascular injury biomarkers are expressed as mean ± *SD*. Representative Flow Cytometry graphics CD45/CD34/CD184 are shown.

Abbreviations: BMI, Body Mass Index; Coro, Coronary Blood; IL‐1β, Interleukin 1 beta; MMP‐9, Matrix Metalloproteinase 9; Periph, Peripheral blood; sICAM‐1, soluble InterCellular Adhesion Molecule 1.

a indicates *P* < 0.05 difference biomarkers from coronary blood vs peripheral circulation.

b indicates *P* < 0.05, w/o MACEs vs MACEs; one‐tail, independent *t* test.

MACEs occurred in 11 (21.1%) patients. One patient (1.9%) died, whereas six patients (11.5%) presented due to angina, requiring coronary angiography and two patients (3.8%) showed myocardial infarction. Four patients (7.7%) attended due to symptoms or evidence of heart failure. Lower coronary concentration of most MPCs was observed in patients who developed MACEs according to sub‐group analysis (Table 1, middle panel), with a more significant decrease of MPCs subpopulations CD34^+^CD133^+^, CD45^+^CD34^+^CD133^+^CD184^+^ and CD45^+^CD34^+^KDR^+^. Likewise, the affected patients showed increased coronary amounts of sICAM‐1 and lower MMP‐9 (Table 1, lower panel). At peripheral blood, we observed the increase in malondialdehyde accompanied by the reduction of superoxide dismutase.

**Figure 1:**
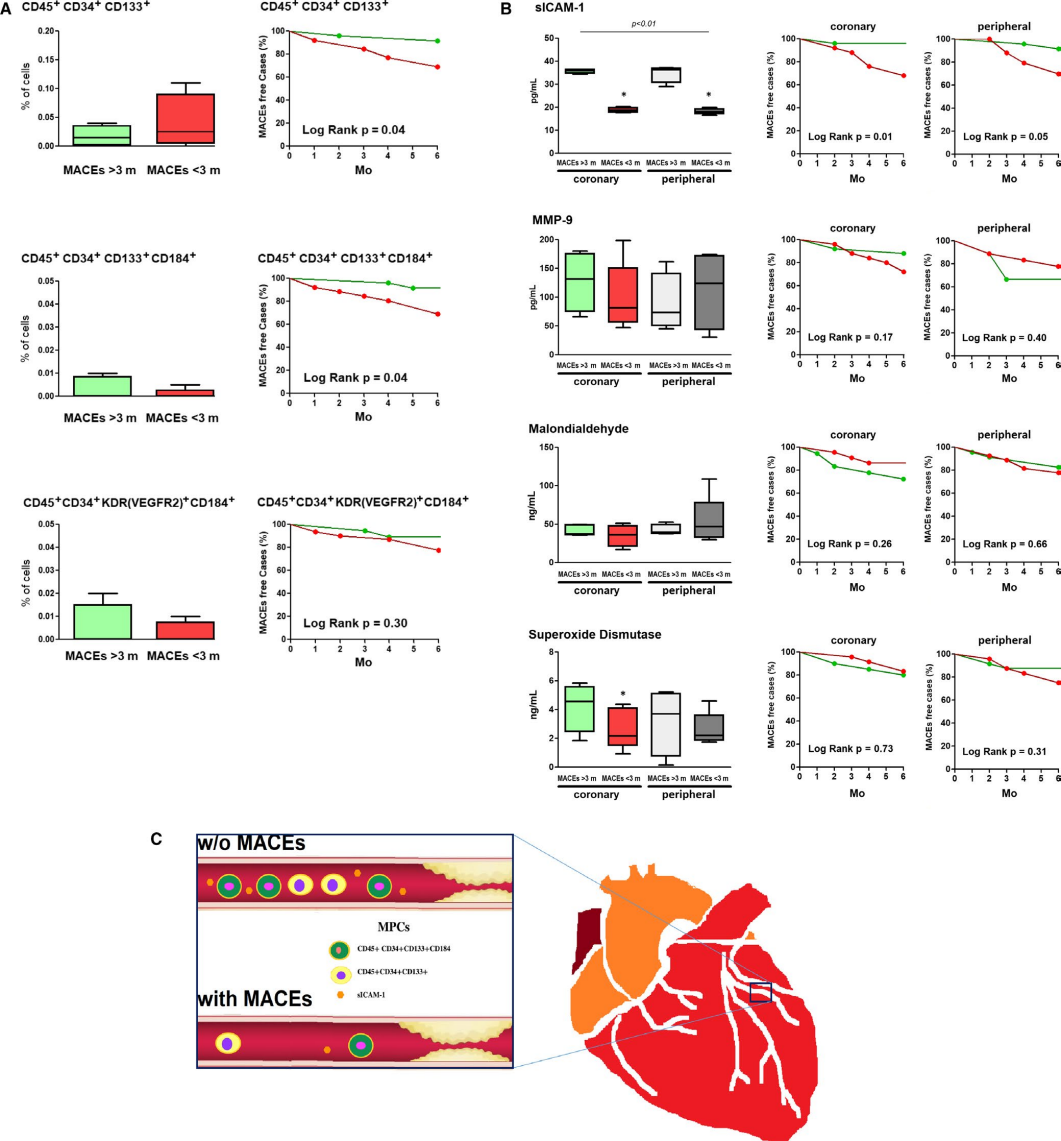
Coronary circulating MPCs, injury vascular mediators and prognosis of MACEs. The figure shows time‐to‐MACEs analysis (box and whisker plots); as well as the MACEs‐free survival (Kaplan‐Meyer plots) corresponding to most associated: A, Coronary MPCs subpopulations and B, Injury vascular mediators. For survival analysis, median values of MPCs or injury vascular mediators were used to dichotomize the population and to compare their survival curves of MACEs during the 6 months follow up. C, Proposed schematic conceptual model of MPCs and soluble mediators related to MACEs. Abbreviatures: MPCs, Mononuclear Progenitor Cells; MACEs, Major Adverse Cardiovascular Events; m, months; sICAM‐1, soluble InterCellular Adhesion Molecule 1; MMP‐9, Matrix Metalloproteinase 9

The study population was further grouped according to time‐to‐MACEs period; where not MPCs (Figure 1A), but lower coronary values of sICAM‐1 and superoxide dismutase (Figure 1B) showed prognostic ability for earlier MACEs; whereas lower coronary MPCs (CD45^+^CD34^+^CD133^+^and CD45^+^CD34^+^CD133^+^CD184^+^) and higher concentration of sICAM‐1 (dichotomized by the median values) demonstrated prognostic ability for MACEs‐free survival (Figure 1A and B, Kaplan‐Meier). Peripheral blood biomarkers showed less significant prognostic performance.

## 4 DISCUSSION

The translational relevance of this study is highlighted by the role of coronary biomarkers related with vascular injury and repair, in the prediction of MACEs and their influence in the prognosis of patients with CAD undergoing angioplasty.

Mobilization of MPCs and increase in adhesion molecules have been observed in acute cardiovascular events.^1^ Moreover, endothelial progenitors exhibit a higher response than endothelial cells,^1^ probably associated with the regenerative process. Circulating mononuclear derived progenitor cells have been related to dysmetabolic biomarkers like osteoprotegerin 11; suggesting its potential to reflect cardiometabolic risk. Particularly, CD45^+^CD34^+^MPCs population have shown their role as useful biomarkers of cardiovascular diseases. Berezin A. et al reported that lower CD45^+^CD34^+^MPCs number in peripheral blood accompanies progression of heart failure severity; independently from clinical demographical characteristics, whereas no difference of CD45^+^CD34^+^MPCs was observed between healthy and CAD populations.^12^

We observed lower MPCs in the population that presented MACEs, with more significant reduction in coronary CD45^+^CD34^+^CD133^+^and CD45^+^CD34^+^CD133^+^CD184^+^ MPCs than other subpopulations. Clinical implication of the role of circulating progenitor cells has been highlighted by well‐designed meta‐analysis of 21 studies; which concludes that lower levels of circulating progenitor cells, particularly those from hematopoietic origin (CD34^+^CD133^+^, RR 2.61 [1.44‐4.74]), are significantly associated with the occurrence of cardiovascular events after coronary intervention. This observation is consistent with our findings; whereas further clinical relevance of progenitor cells in the mentioned meta‐analysis is suggested by risk attenuation in cases of acute heart ischemia and stent re‐stenosis.^13^

The role of soluble adhesion molecules as predictors of MACE and further adverse outcomes in patients submitted to PCI has been previously hypothesized.^7^ Our finding of prognostic ability of sICAM‐1 and MMP‐9 confirms the clinical usefulness of biomarkers of inflammation and vascular repair in the outcomes of patients with ischaemic heart disease submitted to PCI.

In both cases, coronary circulating MPCs and soluble mediators of vascular injury, showed higher prognostic ability than their peripheral distribution. Only few studies have evaluated the potential of coronary sampling for heart diseases.^4^, ^5^ Consistently with our results, interesting differences with peripheral sampling and more precise predictive performance of coronary biomarkers have been evidenced; suggesting the relevance of sampling location in the evaluation of prognostic biomarkers in CAD. To our knowledge, this is the first study prospectively evaluating coronary and peripheral circulating MPCs and soluble mediators, and their role as prognostic biomarkers in population with CAD submitted to coronary angioplasty and stenting.

Peripheral blood pro‐oxidative tendency found in patients with MACEs, denoted by the increase in MDA and decrease in superoxide dismutase, may favour toxic effects leading to MPCs impaired function and accelerated cell death, as has been suggested.^14^ Lower MPCs accompanied by lower MMP‐9 may affect intracoronary angio‐repair ability and adequate endothelial remodelling after PCI, which may lead to MACEs.

Despite limitations like the low number of patients, short time follow‐up and heterogeneity of indications for coronary angiography, this prospective study strongly suggest that coronary concentration of biomarkers related with vascular repair, such as MPCs subpopulations and adhesion molecules, may predict MACEs and impact prognosis in patients with CAD undergoing angioplasty.

## CONFLICT OF INTEREST

The authors confirm that there is no conflict of interest.

## AUTHOR CONTRIBUTIONS

*Design and results analysis*: JA Suárez‐Cuenca; AS Ruíz‐Hernández and LJ Díaz‐Hernández. *Experimental Assays*: E. Vera‐Gómez; A. Hernández‐Patricio and A. Contreras‐Ramos. *Sample collection*: R. Robledo‐Nolasco; MA Alcántara‐Meléndez; KS Sánchez‐Díaz and JA Buendía‐Gutiérrez. *Critical Manuscript Review*: P. Mondragón‐Terán P and R. Pérez‐Cabeza de Vaca.
